# Supplementary material for: Reporting Standards for a Bland–Altman Agreement Analysis: A Review of Methodological Reviews
Source: Diagnostics (Basel). 2020 May 22;10(5):334. doi: 10.3390/diagnostics10050334 (PMC7278016; doi:10.3390/diagnostics10050334)
Supplement: Supplementary file 1 [file diagnostics-10-00334-s001.zip › Supplemental Data 4.pdf]

```
                                                     ___  ____  ____  ____  ____(R)
                                                    /__    /   ____/   /   ____/
                                                   ___/   /   /___/   /   /___/
                                                     Statistics/Data Analysis

                                                       User: Supplemental Data 4

1 . do "C:\Users\R60\AppData\Local\Temp\STD00000000.tmp"

2 . import delimited using "Supplemental Data 2.csv", clear
  (4 vars, 420 obs)

3 . mixed value i.repeat || _all:R.patid || _all:R.rater , reml

  Performing EM optimization:

  Performing gradient-based optimization:

  Iteration 0:   log restricted-likelihood = -931.56022
  Iteration 1:   log restricted-likelihood = -931.56022

  Computing standard errors:

  Mixed-effects REML regression                   Number of obs     =        420
  Group variable: _all                            Number of groups  =          1

                                                  Obs per group:
                                                                min =        420
                                                                avg =      420.0
                                                                max =        420

                                                  Wald chi2(1)      =      24.32
  Log restricted-likelihood = -931.56022          Prob > chi2       =     0.0000

  ------------------------------------------------------------------------------
         value |      Coef.   Std. Err.      z    P>|z|     [95% Conf. Interval]
  -------------+----------------------------------------------------------------
      2.repeat |  -.5889469   .1194229    -4.93   0.000    -.8230115   -.3548822
         _cons |   24.33453   .5292671    45.98   0.000     23.29719    25.37188
  ------------------------------------------------------------------------------

  ------------------------------------------------------------------------------
    Random-effects Parameters  |   Estimate   Std. Err.     [95% Conf. Interval]
  -----------------------------+------------------------------------------------
  _all: Identity               |
                  var(R.patid) |   35.99358   4.360442      28.38613    45.63981
  -----------------------------+------------------------------------------------
  _all: Identity               |
                  var(R.rater) |   .1400625    .116635      .0273839    .7163873
  -----------------------------+------------------------------------------------
                 var(Residual) |   1.019588   .0875645      .8616319    1.206502
  ------------------------------------------------------------------------------
  LR test vs. linear model: chi2(2) = 843.54               Prob > chi2 = 0.0000

  Note: LR test is conservative and provided only for reference.

4 . di 2.77*sqrt(1.019588)
  2.7969978

5 . di 2.77*sqrt(1.019588  + .1400625)
  2.9829318

6 .
  end of do-file

7 .
```
