# Supplementary material for: Reporting Standards for a Bland–Altman Agreement Analysis: A Review of Methodological Reviews
Source: Diagnostics (Basel). 2020 May 22;10(5):334. doi: 10.3390/diagnostics10050334 (PMC7278016; doi:10.3390/diagnostics10050334)
Supplement: Supplementary file 1 [file diagnostics-10-00334-s001.zip › Supplemental Table 3.pdf]

Supplemental Table 3: Reasons for exclusions

Search item no.	Article	Reason for exclusion
228	Walter SR, Dunsmuir WTM, Westbrook JI. Inter-observer agreement and reliability assessment for observational studies of clinical work. J Biomed Inform. 2019 Dec;100:103317. doi: 10.1016/j.jbi.2019.103317.	Reliability (iota score; kappa)
764	Gerke O, Möller S, Debrabant B, Halekoh U; Odense Agreement Working Group. Experience Applying the Guidelines for Reporting Reliability and Agreement Studies (GRRAS) Indicated Five Questions Should Be Addressed in the Planning Phase from a Statistical Point of View. Diagnostics (Basel). 2018 Sep 24;8(4). pii: E69. doi: 10.3390/diagnostics8040069.	GRRAS (comment)
1170	Dixon PM, Saint-Maurice PF, Kim Y, Hibbing P, Bai Y, Welk GJ. A Primer on the Use of Equivalence Testing for Evaluating Measurement Agreement. Med Sci Sports Exerc. 2018 Apr;50(4):837-845. doi: 10.1249/MSS.0000000000001481.	Methodology (alternative to BA LoA: bioequivalence testing)
1546	Barth J, de Boer WE, Busse JW, Hoving JL, Kedzia S, Couban R, Fischer K, von Allmen DY, Spanjer J, Kunz R. Inter-rater agreement in evaluation of disability: systematic review of reproducibility studies. BMJ. 2017 Jan 25;356:j14. doi: 10.1136/bmj.j14.	Reliability (intraclass correlation coefficient (ICC))
1768	Farzin B, Gentric JC, Pham M, Tremblay-Paquet S, Brosseau L, Roy C, Jamali S, Chagnon M, Darsaut TE, Guilbert F, Naggara O, Raymond J. Agreement studies in radiology research. Diagn Interv Imaging. 2017 Mar;98(3):227-233. doi: 10.1016/j.diii.2016.05.014.	GRRAS (application; systematic review)
2215	Dhillon SS, Sima CA, Kirkham AR, Syed N, Camp PG. Physical Activity Measurement Accuracy in Individuals With Chronic Lung Disease: A Systematic Review With Meta-Analysis of Method Comparison Studies. Arch Phys Med Rehabil. 2015 Nov;96(11):2079-88.e10. doi: 10.1016/j.apmr.2015.05.015.	Meta-analysis on quantitative outcome measures
2331	Carkeet A. Exact parametric confidence intervals for Bland-Altman limits of agreement. Optom Vis Sci. 2015 Mar;92(3):e71-80. doi: 10.1097/OPX.0000000000000513.	Methodology (confidence intervals for BA LoA)
2493	Xu S, Lorber MF. Interrater agreement statistics with skewed data: evaluation of alternatives to Cohen's kappa. J Consult Clin Psychol. 2014 Dec;82(6):1219-27. doi: 10.1037/a0037489.	Methodology (alternatives to kappa)
2976	Oster NV, Carney PA, Allison KH, Weaver DL, Reisch LM, Longton G, Onega T, Pepe M, Geller BM, Nelson HD, Ross TR, Tosteson AN, Elmore JG. Development of a diagnostic test set to assess agreement in breast pathology: practical application of the Guidelines for Reporting Reliability and Agreement Studies (GRRAS). BMC Womens Health. 2013 Feb 5;13:3. doi: 10.1186/1472-6874-13-3.	GRRAS (application)
3137	Hoy D, Brooks P, Woolf A, Blyth F, March L, Bain C, Baker P, Smith E, Buchbinder R. Assessing risk of bias in prevalence studies: modification of an existing tool and evidence of interrater agreement. J Clin Epidemiol. 2012 Sep;65(9):934-9. doi: 10.1016/j.jclinepi.2011.11.014.	Methodology (assessment of risk of study bias)
3391	Jones M, Dobson A, O'Brian S. A graphical method for	Methodology (graphical

	assessing agreement with the mean between multiple observers using continuous measures. Int J Epidemiol. 2011 Oct;40(5):1308-13. doi: 10.1093/ije/dyr109.	assessment of agreement of more than two observers in quantitative outcomes)
3442 (3471, 3527)	Kottner J, Audige L, Brorson S, Donner A, Gajewski BJ, Hróbjartsson A, Roberts C, Shoukri M, Streiner DL. Guidelines for Reporting Reliability and Agreement Studies (GRRAS) were proposed. Int J Nurs Stud. 2011 Jun;48(6):661-71. doi: 10.1016/j.ijnurstu.2011.01.016.	GRRAS (guideline proposal)
3679	Jacobs PC, Prokop M, Oen AL, van der Graaf Y, Grobbee DE, Mali WP. Semiquantitative assessment of cardiovascular disease markers in multislice computed tomography of the chest: interobserver and intraobserver agreements. J Comput Assist Tomogr. 2010 Mar-Apr;34(2):279-84. doi: 10.1097/RCT.0b013e3181bbcff6.	Reliability (weighted kappa, ICC)
3904	Cecconi M, Rhodes A, Poloniecki J, Della Rocca G, Grounds RM. Bench-to-bedside review: the importance of the precision of the reference technique in method comparison studies--with specific reference to the measurement of cardiac output. Crit Care. 2009;13(1):201. doi: 10.1186/cc7129.	Methodology (30% in the percentage error; worked example)
4394	Ouwendijk R, Kock MC, Visser K, Pattynama PM, de Haan MW, Hunink MG. Interobserver agreement for the interpretation of contrast-enhanced 3D MR angiography and MDCT angiography in peripheral arterial disease. AJR Am J Roentgenol. 2005 Nov;185(5):1261-7.	Reliability (weighted kappa)
4567	Gornstein B, Jacobs T, Bédard Y, Biscotti C, Ducatman B, Layfield L, McKee G, Sneige N, Wang H. Interobserver agreement of a probabilistic approach to reporting breast fine-needle aspirations on ThinPrep. Diagn Cytopathol. 2004 Jun;30(6):389-95.	Methodology (assessment of probabilistic approach by kappa and Spearman's rho)
4763	Stockbridge HL, Lewis D, Eisenberg B, Lee M, Schacher S, van Belle G, Keifer M, Brodkin CA, Buchwald D. Brain SPECT: a controlled, blinded assessment of intra-reader and inter-reader agreement. Nucl Med Commun. 2002 Jun;23(6):537-44.	Reliability (per cent kappa agreement)
5050	Plebani M, Bernardi D, Basso D, Borghesan F, Faggian D. Measurement of specific immunoglobulin E: intermethod comparison and standardization. Clin Chem. 1998 Sep;44(9):1974-9.	Methodology (comparison of the analytical and clinical performances of four second-generation techniques for allergen-specific IgE measurement in serum)
5097	Nevill AM, Atkinson G. Assessing agreement between measurements recorded on a ratio scale in sports medicine and sports science. Br J Sports Med. 1997 Dec;31(4):314-8.	Methodology (assessment of heteroscedasticity across 13 studies)
5178	Carr JE, Austin J, Hatfield DB, Bailey JS. The standard deviation as an informative measure of variability in reporting interobserver agreement means. J Behav Ther Exp Psychiatry. 1996 Sep;27(3):263-7.	Methodology (standard deviation as supplement to mean in terms of spread in the data)
5458	Becker MP. Using association models to analyse agreement data: two examples. Stat Med. 1989 Oct;8(10):1199-207.	Methodology (alternative to BA LoA: association models)
