# Supplementary material for: Reporting Standards for a Bland–Altman Agreement Analysis: A Review of Methodological Reviews
Source: Diagnostics (Basel). 2020 May 22;10(5):334. doi: 10.3390/diagnostics10050334 (PMC7278016; doi:10.3390/diagnostics10050334)
Supplement: Supplementary file 1 [file diagnostics-10-00334-s001.zip › Supplemental Data 1.pdf]

Supplemental Data 1: Details for search conducted with MEDLINE/PubMed

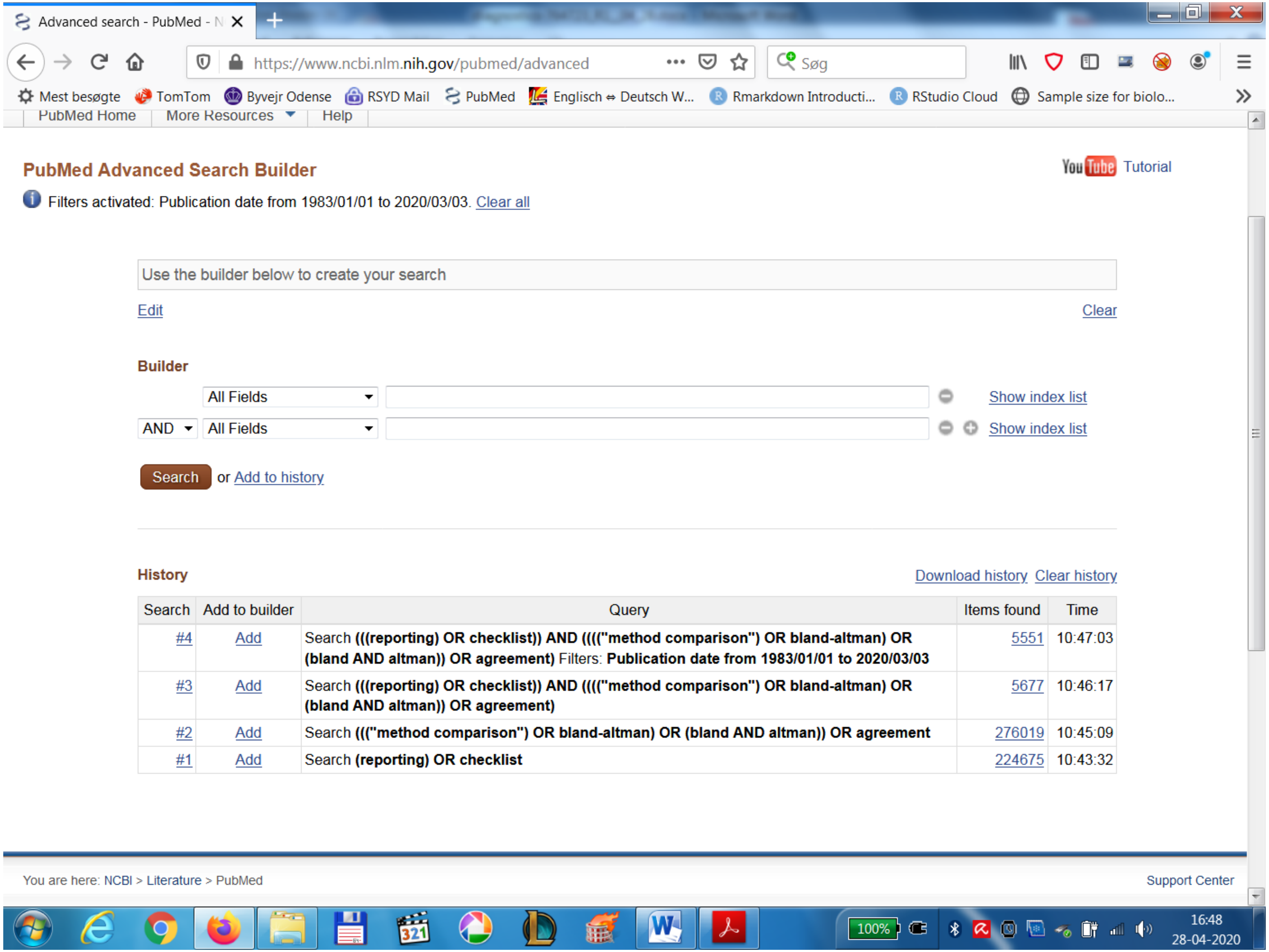

Advanced search - PubMed - N

https://www.ncbi.nlm.nih.gov/pubmed/advanced

PubMed Home | More Resources | Help

PubMed Advanced Search Builder

YouTube Tutorial

Filters activated: Publication date from 1983/01/01 to 2020/03/03. Clear all

Use the builder below to create your search

Edit | Clear

Builder

All Fields | Show index list

AND | All Fields | Show index list

Search or Add to history

History

Download history | Clear history

Search	Add to builder	Query	Items found	Time
#4	Add	Search **(((reporting) OR checklist)) AND ((((("method comparison") OR bland-altman) OR (bland AND altman)) OR agreement)** Filters: **Publication date from 1983/01/01 to 2020/03/03**	5551	10:47:03
#3	Add	Search **(((reporting) OR checklist)) AND ((((("method comparison") OR bland-altman) OR (bland AND altman)) OR agreement)**	5677	10:46:17
#2	Add	Search **((("method comparison") OR bland-altman) OR (bland AND altman)) OR agreement**	276019	10:45:09
#1	Add	Search **(reporting) OR checklist**	224675	10:43:32

You are here: NCBI > Literature > PubMed | Support Center

16:48 28-04-2020
